# “I don’t think education is the answer”: A corpus-assisted ecolinguistic analysis of plastics discourses in the UK

**DOI:** 10.1515/jwl-2022-0017

**Published:** 2022-08-15

**Authors:** Emma Franklin, Joanna Gavins, Seth Mehl

**Affiliations:** Research Group in Computational Linguistics, University of Wolverhampton, Wolverhampton, UK; School of English, University of Sheffield, Sheffield, UK; Digital Humanities Institute, University of Sheffield, Sheffield, UK

**Keywords:** cognitive linguistics, corpus linguistics, ecolinguistics, packaging, plastic

## Abstract

Ecosystems around the world are becoming engulfed in single-use plastics, the majority of which come from plastic packaging. Reusable plastic packaging systems have been proposed in response to this plastic waste crisis, but uptake of such systems in the UK is still very low. This article draws on a thematic corpus of 5.6 million words of UK English around plastics, packaging, reuse, and recycling to examine consumer attitudes towards plastic (re)use. Utilizing methods and insights from ecolinguistics, corpus linguistics, and cognitive linguistics, this article assesses to what degree consumer language differs from that of public-facing bodies such as supermarkets and government entities. A predefined ecosophy, prioritizing protection, rights, systems thinking, and fairness, is used to not only critically evaluate narratives in plastics discourse but also to recommend strategies for more effective and ecologically beneficial communications around plastics and reuse. This article recommends the adoption of ecosophy in multidisciplinary project teams, and argues that ecosophies are conducive to transparent and reproducible discourse analysis. The analysis also suggests that in order to make meaningful change in packaging reuse behaviors, it is highly likely that deeply ingrained cultural stories around power, rights, and responsibilities will need to be directly challenged.

## 1 Introduction

Single-use plastics account for roughly half of all plastics produced and thrown away (Geyer et al. 2017), and almost 70% of plastic waste is plastic packaging (WRAP 2021b). It is estimated that there are currently around eight billion tons of plastic in the Earth’s system, and that roughly ten million tons end up in our oceans every year (Thompson et al. 2004). By 2050, it is projected that there will be as much as 40 billion tons of plastic in circulation on our planet (Geyer et al. 2017). While single-use plastic has enabled countless life-saving and life-aiding interventions, for humans in particular, it has simultaneously become enmeshed in a global pollution and consumption crisis that negatively affects all life on Earth. In this article, we report the process and outcome of an ecolinguistics-informed, corpus-assisted discourse analysis of the language of plastic packaging, disposal, and (re)use. This was conducted as part of *Many Happy Returns* (MHR), a large, multidisciplinary project funded by UK Research and Innovation (UKRI), the aim of which is to develop reusable packaging systems in an effort to reduce reliance on single-use plastics in the UK.

This article is structured as follows. In the remainder of this section, we provide some background to the problem under investigation (the use of plastics, in particular single-use plastics), and a brief rationale for researchers taking an ecolinguistic approach, or at the very least adopting ecolinguistic principles, in addressing this problem. Section 2 describes the research context of our own (eco)linguistic work, first within the broader context of the existing literature and then within our multidisciplinary research project environment. Next, in Section 3, we outline the methodology of our study, starting with our ecosophy and then describing in detail the means by which we gathered our data and analyzed it using corpus linguistic software, incorporating cognitive linguistic and ecolinguistic perspectives. Section 4 reports some of the findings we were able to achieve using this approach, and Section 5 offers some recommendations for practical applications of these linguistic findings. Finally, in Section 6, we conclude the article with some of the potential implications of this work, as well as some suggestions for future research.

### 1.1 Plastics: A complex picture

Most notably hailed as a marvel of convenience in the 1950s (e.g. Life 1955), plastic is now perhaps better known for its polluting capabilities and its ubiquity as a form of ever-fragmenting waste. Its abundance and durability, which have made it a wonderfully cheap and reliable construction material for humans, have also endowed it with a lasting presence in ecosystems all around the world; some plastic has already occupied natural environments for decades beyond its original, typically fleeting, use (Lebreton et al. 2018). The long-term environmental impacts of plastic are claimed to be, at best, “unknown” and “irreversible” (Hohn et al. 2020), while other predictions are much more sobering: that plastic fragmentation is the beginning of a delayed, cumulative release of toxic chemicals, a “global plastic toxicity debt” (Rillig et al. 2021); and that plastic is “poised to dominate the 21st century as one of the yet-unchecked drivers of climate change” (Altman 2022).

However, one also cannot overstate the usefulness of plastic. Plastics enable cheap, readily available infrastructure that delivers life-sustaining water to billions of people in the form of pipelines, for example, as well as all kinds of food products in lightweight, transportable containers that can effectively preserve foods and reduce food waste; they facilitate sterile, affordable, and safe medical equipment in the form of catheters, sheets, syringes, protective packaging, and personal protective equipment; and there is an almost endless list of basic-necessity artifacts such as clothing, vehicles, technology, and affordable housing and building materials that are dependent on their plastic constituents (George 2020). Due to its complex enmeshment in global networks of production and consumption, plastic has become practically irreplaceable in many contexts. Not only is it physically enduring, but socially, culturally, and economically, too (see Evans et al. 2020).

To further complicate matters, plastic is not a straightforward material to dispose of responsibly. A range of different polymers are used and often combined, along with chemical additives, to make different kinds of plastics with specific material properties, and not all of these plastics can effectively be recycled (Bucknall 2020). Even those which theoretically could be recycled are often not, due to local logistical and economical constraints (Bucknall 2020). Citizens commonly report difficulty in knowing which items are recyclable – in the UK, this can vary even from one city to the next – and indeed cannot know for certain whether their efforts will ensure that the waste is eventually recycled (INCPEN 2021). Even when plastics are successfully recycled, the quality of the plastic is reduced with each iteration. This depends crucially on the type of plastic; for example, 100% recycled polyethylene terephthalate (PET) can be used in a closed loop if the plastic is upgraded at each stage, whereas polyolefins (polyethylene and polypropylene) need the addition of virgin polymer at every recycling event so that properties are maintained (see Eriksen et al. 2019). A life-cycle assessment, the calculation of all of the environmental impacts associated with a product, from its manufacturing to its disposal, may also rule out recycling as the most ecological option for a particular kind of plastic waste product (Walker and Rothman 2020). Beyond all of these material limitations, the story that recycling promotes – that single-use products are largely redeemable, and that disposing of them is unproblematic – does not serve to challenge our over-production and over-consumption of single-use plastics and plastic packaging. In short, recycling is not enough.

Rather than aiming to entirely avoid or simply recycle plastics, reusable plastic packaging systems are increasingly being looked to as a potential solution to plastic waste (Greenwood et al. 2021); a circular economy approach has the potential to reduce the yearly volume of plastics entering the oceans by 80% and to reduce greenhouse gas emissions by 25% (Woolven 2021). While reusable packaging may not be suitable for all products, there is a wide range of goods that have the potential to be provided on a large-scale refill or return basis. The “refill” option of such schemes normally involves taking one’s own reusable container to a store and filling it, or taking home a minimally-packaged product to refill one’s home container. The “return” option normally involves receiving a pre-packaged product and then returning the reusable container to the vendor or manufacturer, or having it collected. “Reuse” is sometimes also considered to include repurposing (i.e. buying a single-use product and later reusing it for a different purpose), but within our project, *Many Happy Returns*, packaging is considered to be strictly “reusable” only when it can be used more than once for the same purpose, i.e. through being refillable or returnable.

### 1.2 Linguistic intervention

In addition to technological innovation, achieving system change requires a sensitivity to public sentiment as well as the ability to communicate complex concepts in ways that will make sense to many people. This means observing, on a large scale, the ways members of the public express their views about plastics, disposal, and reuse, and then developing communication strategies that align with (or where necessary, challenge) the most prominent and pervasive attitudes towards plastic (re)use. Encouraging everyday behavior change on a large scale involves an uncovering – and reframing – of the cultural *stories* (Stibbe 2021b) underpinning behavior. These are not the stories that are “read to children at bedtime, shared around a fire, or conveyed through anecdotes in formal speeches”, but “cognitive structures in the minds of individuals which influence how they think, talk and act” (Stibbe 2020: 2). For this reason, it is essential not only that analysis of the discourse used around plastics is based on the systematic examination of large, representative linguistic datasets, but that it is further underpinned by an understanding of language that aligns with the most up-to-date research in the cognitive sciences. Such an understanding is provided by cognitive linguistics, which forms a further important component of our theoretical approach.

The way we frame complex environmental issues is especially critical, according to Lakoff (2010). “Facts”, he argues, “must make sense in terms of their system of frames, or they will be ignored […] to understand something complex, a person must have a system of frames in place that can make sense of the facts” (Lakoff 2010: 73). We understand “facts” here to refer to any kind of information that is conveyed in the interest of public science communication. If we are to help laypersons, i.e. most members of the public, to properly understand the complex nature of plastic and its correct use and disposal, linguistic reframing strategies must be deployed with a sensitivity to the stories and frames that already exist in the minds of many people.

Ecolinguistics, then, which “seeks to explore linguistic phenomena found in inter-language, inter-human, and human-nature relationships from the perspective of ecological philosophy” (Chen 2016: 109), represents an ideal means of unravelling plastic’s complexities, both as a material in our society and as stories in the minds of plastic users. We maintain that only through (i) careful and critical application of ecolinguistic principles, while (ii) drawing on cognitive linguistic expertise, and (iii) basing findings on large and representative samples of linguistic data, will it be possible to craft new, empirically reliable stories that help to encourage a more mindful relationship with plastic products. Of course, linguistic research is just one piece of the plastics puzzle, but it is a crucial one.

## 2 Research context

### 2.1 Science communication

Faced with complex technical and environmental problems, and in a so-called “post-truth world”, effective science communication is now recognized as being more important than ever (Kopf et al. 2019). Science communication can take a diverse range of forms, from documentary films and informational flyers to stand-up comedy, even (Bowater and Yeoman 2012), and its practitioners are increasingly being referred to as “knowledge brokers” (Meyer 2010; cf. Pielke 2007) or “knowledge translators” (Bielak et al. 2008) in an effort to emphasize the co-creative and dialogic nature of good science communication. Regardless of issue or medium, it is argued that public-facing communications ought to employ listening as well as speaking, and should engage with a wide range of audiences in ways that are tailored for each audience (Bielak et al. 2008). This requires first doing empirical research to understand what laypersons already know, as well as recognizing that communication about science “does not occur in a vacuum” (Fischhoff and Scheufele 2013: 1); science communication is, in itself, a form of political communication (Scheufele 2014).

Progressive public communications in the UK have been championed perhaps most notably by the Public Interest Research Centre (PIRC),1
https://publicinterest.org.uk/ (accessed 6 May 2021). whose framing and communication toolkits have spanned topics such as nature, equality, climate justice, and the economy. PIRC work with community interest groups as well as other framing specialists such as the FrameWorks Institute2
https://www.frameworksinstitute.org/ (accessed 6 May 2021). to evaluate how complex issues such as the environment and the economy are understood by members of the British public, and then to produce co-created booklets of framing and narrative recommendations for a range of communicators. They also disseminate their findings in the form of posters, card decks, and postcards; their ‘The Narratives We Need’ postcard, for example, distils the findings of 15 research projects and ten years of work into five key questions for public-issue communicators: (i) “Are you talking to people’s best selves?”; (ii) “Are you showcasing a big, diverse ‘us’?”; (iii) “Are you showing we can all play a role in change?”; (iv) Are you highlighting how the system is unfairly designed?”; and (v) “Are you demonstrating that change is possible?” (PIRC 2020). Along with the FrameWorks Institute and the US-based Center for Story-Based Strategy,3
https://www.storybasedstrategy.org/ (accessed 6 May 2021). PIRC represents a leading example of politically sensitive, narrative-driven, public-issue communications, or knowledge brokering. Similar approaches are gradually being adopted, based on more rigorous methodological foundations, by academics working with metaphor and narrative: an output of Semino et al.’s (2017) work on cancer discourses was an illustrated “metaphor menu”4
http://wp.lancs.ac.uk/melc/the-metaphor-menu/ (accessed 6 May 2021). for cancer patients and healthcare practitioners to use in cancer-related communications; and Lockton et al. (2019) have translated their design research into a toolkit and a deck of cards that can be used in metaphor-generating workshops, for example.

Where pro-environmental communication is concerned, a growing amount of research is being carried out by marketing and business scholars, in particular, on optimal ways of promoting environmentally beneficial products and practices (Taylor 2015). Despite often having a somewhat capitalistic undercurrent, industry-oriented research on “green” marketing and advertising has produced many useful, experimental findings around effective use of language, particularly with regard to promoting pro-environmental behavior change. Studies on the appropriate level of assertiveness, for example, have found that gentler and less assertive phrasing is typically found to yield more compliance on a range of issues, including environmental practices (Baek et al. 2015), and that employing a greater number of “green” messages can in fact backfire, leading to reactance (Moyer-Gusé et al. 2019) and negative brand perception (Olsen et al. 2014). There are a few instances in which more forceful messaging may be more effective, however: when the recipient already invests some effort in the promoted behavior (Baek et al. 2015); when the issue is accepted to be of extreme importance (Kronrod et al. 2012b); or when the message encourages “hedonic consumption” rather than virtuous behaviors (Kronrod et al. 2012a). Interestingly, and perhaps unsurprisingly, an experimental study on the language of returnable packaging labels found that “ease of use” messages, as opposed to messages that justify why returning packaging is important, were more effective at raising consumers’ intentions to actively participate in return schemes (Ratnichkina et al. 2021). A key finding of WRAP (2021a), however, is that consumers typically spend very little time reading such labels – around ten seconds or less – and as such they must be clear, simple, direct, and easy to find if they are to have any benefit.

### 2.2 Strategic use of framing

A reasonably consistent finding across messaging studies is that negatively framed messages tend to be more effective than positively framed ones for influencing attitudes and behaviors (Amatulli et al. 2019; Davis 1995; Ganzach and Karsahi 1995; Levin et al. 1998; Meyerowitz and Chaiken 1987; Olsen et al. 2014; White et al. 2011). That is to say, messages that emphasize the benefits of engaging in pro-environmental behavior are typically less persuasive than those that emphasize the risks or potential losses associated with not engaging in that behavior, e.g. the benefits of purchasing a ‘green’ product versus the disadvantages associated with not purchasing it, or positively framed product claims such as “biodegradable” and “recyclable” versus negatively framed ones such as “no pesticides” and “no phosphates” (Olsen et al. 2014). Amatulli et al. (2019) argue that this effect can be attributed specifically to the activation of the emotion of shame, or “anticipated shame”, while White et al. (2011) propose that it is due in part to fluency, i.e. ease of cognitive processing. White et al. (2011) contest, however, that negative frames are always more effective: they find that negative, or loss-framed, messages are more persuasive in contexts of low-level or concrete actions (e.g. the act of reusing or returning packaging), while positive, or gain-framed, messages are potentially more appropriate for high-level or abstract purposes (e.g. fostering beliefs about why reusing and returning packaging is important). These are also the findings of Lord (1994), whose study on recycling concludes that the most effective motivator of recycling behaviors is a negatively framed message conveyed by a personal acquaintance, while positively framed messages are more effective at promoting a favourable attitude towards recycling generally.

All of these findings align with research in cognitive linguistics, which broadly agrees on a view of linguistic negation of any kind as having a fundamentally foregrounding effect (see Hidalgo Downing [2000] for a useful overview). Givón (1993) explains that this is because negation essentially reverses the normative means through which human beings understand stasis as a conceptual background, against which descriptions of events or change stand out in our minds. Givón points out that there is a predominance of language in everyday communication which fits this standard conceptual structure: events are construed through language as more informative than non-events. This predominance also affects how we perceive departures from it made through negation, where the event, rather than inertia, is established as the ground and a non-event becomes more salient and more informative (Givón 1993: 190). When negation is used in language, therefore, it is discoursally and conceptually deviant, drawing attention both to itself as a linguistic form and to the non-events it describes.

Negation also acts to defeat our expectations in the discourse-world of a particular communication and generates implicature as a result. Nahajec (2009: 110) explains that “in order to deny a prior proposition, implicit or explicit, we have to conceptualize or create a mental representation of what is being denied”; in Olsen et al.’s (2014) examples of labels like “no phosphates” above, then, readers of these messages must first mentally represent an affirmative situation in which the product *does* contain phosphates, in order to then understand the reverse of that state of affairs. The label also carries a set of presuppositions and implicatures, including some assumption that the product ordinarily might be expected to, or even *should* contain phosphates, but does not. Cognitive linguistic research on negation thus enables us to understand the potential rhetorical power of negatively framed messaging, which places additional demands on our conceptual processing, challenges our expectations and presuppositions, and has an overall disruptive pragmatic effect in discourse.

### 2.3 Linguistic approaches

Cognitive linguistic studies, drawing on both psychology and narrative theory, provide supporting evidence for the benefits of a narrative-based approach, both to the communication of scientific research and to the creation and nurturing of movements for social change. Perhaps most notably in recent years, we have seen the rise of emotive, storytelling documentaries that have succeeded in motivating public and even policy discussions around environmental concerns (Nolan et al. 2022), a phenomenon commonly dubbed the “Blue Planet effect” (Gell 2019). The powerful role such storytelling can play in creating or encouraging mass social movements and systemic change is well attested in cognitive accounts of narrative persuasion (see Green et al. 2002). Jacobs (2002), for instance, argues that narrative structures are instrumental in the forging of collective social identities and also allow members of those collectives to be furnished with a set of stories about a shared past, present, and future. Narratives, in essence, allow people to rationalise and order events in a conceptually manageable chronological sequence and to position themselves and others within that sequence in roles such as “hero” or “villain”. As Jacobs puts it, the creation of plot “encourages the public concentration of attention onto specific events, encouraging discussion about the meaning of those events”, while narrative features such as opposing characters “serve to dramatize a society’s deep cultural codes, increasing the likelihood of a continued emotional investment in public life” (Jacobs 2002: 218).

Ecolinguists, not to be confused with language ecologists (Alexander and Stibbe 2014), contribute to our understanding of environmental discourses by critically analyzing instances of language for its pro- and anti-ecological sentiment. Stibbe, especially, has furthered the field of ecological discourse analysis by drawing on cognitive linguistic concepts of story, framing, and metaphor, as well as critical discourse analysis methods (e.g. Fairclough 2001; see Stibbe 2001), to develop a coherent approach to ecolinguistics: a positive, critical, ecological discourse analysis (see Stibbe 2021a, 2021b). Doing ecolinguistics in this sense involves taking an explicit ecological and philosophical stance and then using a range of discourse analytic approaches to determine whether or not a discourse is aligned with that philosophy (see Section 3.1 for more on *ecosophy*). Ecolinguistics has been used to analyze the language of economics textbooks (Stibbe 2020, 2021b), haiku poetry (Stibbe 2007, 2021b), computer games (Poole and Spangler 2020), road users (Caimotto 2020), environmental debates (Poole 2018), climate change news stories (Norton and Hulme 2019), news reports (Cheng and He 2021), and tourism texts (Ponton 2022), to name a handful of recent examples. To our knowledge, it has not yet been used to analyze the language of plastics and plastic packaging, nor has it been used in industry-facing research, e.g. on consumer messaging strategies.

Increasingly, ecolinguistics is also being employed in conjunction with corpus linguistic methods, a survey of which can be found in Poole (2022), the first book-length treatment of corpus-assisted ecolinguistics. Doing linguistics with a *corpus* – literally, a “body” of text – simply means conducting linguistic analysis on a very large, computer-readable text or set of texts and, consequently, employing computational methods in processing and analyzing said text(s). The fact that a corpus can be constructed from any of a wide range of texts (or indeed other modalities), and that there is no prescribed method for doing corpus analysis, means that corpus methods can be applied to a wide range of language data and research questions. Corpus-assisted discourse analysis, for instance, is now commonplace and has been used to explore a host of societal and environmental issues, from media representations of mental health (Hunt and Brookes 2020), Islam (Baker et al. 2013), and feminism (Jaworska and Krishnamurthy 2012), to discourses of nonhuman animals (Sealey and Charles 2013), the environment (Lischinsky 2015), and climate change (Grundmann and Krishnamurthy 2010; Koteyko et al. 2013; Liu and Huang 2022), amongst many others. When it comes to corpus studies on plastic packaging and related themes, we are aware of just two: Napolitano (2018), a corpus-assisted analysis of media discourse around the plastic bag ban in Australia; and Niceforo (2021), a corpus-assisted ecolinguistic discourse analysis of two public-facing reports on plastic pollution.

Corpus linguistics and cognitive linguistics have also been successfully combined, often with corpus data providing an additional testbed for existing cognitive linguistic theories (Grondelaers et al. 2007; see also Arppe et al. [2010] and Gries and Stefanowitsch [2006] for full discussion of the benefits and challenges of combining corpus-linguistic methodologies with cognitive theory). Cognitive linguistics also offers valuable insight into metaphor, instances of which can reliably be observed with the use of a corpus (Semino 2017). The relationship between corpus frequency and specific cognitive processes such as entrenchment (Schmid 2007), however, is not entirely certain, and has been complicated by methodological variation in how researchers measure both corpus frequency and processes including entrenchment (cf. Mehl 2018). Nonetheless, text frequency – one of the main affordances of corpus linguistics – can be interpreted as representing an exposure rate (Wallis 2012), and the more a language user is exposed to a linguistic feature, the more the cognitive activation of that feature ought to become routinized or entrenched (Schmid 2007: 119–120). This claim would apply to the entrenchment of linguistic frames or stories such as those around plastics and reuse.

### 2.4 Language and *Many Happy Returns*

*Many Happy Returns* is a large, multidisciplinary project funded by UK Research and Innovation (UKRI), specifically as part of the UKRI’s Enabling Research competition in its Smart Sustainable Plastic Packaging Challenge. The aims of this funding initiative are “to find solutions to existing issues with plastic packaging, reduce plastic pollution and unlock barriers to create fundamental changes in the industry” (UKRI 2020), and MHR’s proposed contribution, as mentioned in Section 1, is to innovate and encourage reusable plastic packaging systems as a means of reducing single-use plastic waste. To this end, it has five research teams, each covering one of the work packages (Language, Change, Willingness, Technology, and Life Cycle), and represents seven departments (English, Geography, Psychology, Mechanical Engineering, Chemical and Biological Engineering, the Management School, and Chemistry) across four faculties at the University of Sheffield. Beyond the University, the project has external partners ranging from non-profit organizations such as OPRL (On-Pack Recycling Label),5
https://www.oprl.org.uk/ (accessed 6 May 2021). City to Sea,6
https://www.citytosea.org.uk/ (accessed 6 May 2021). and WRAP,7
https://wrap.org.uk/ (accessed 6 May 2021). to major UK supermarkets such as Morrisons, Co-op, and Marks & Spencer, as well as larger, multinational corporations including Nestlé, Unilever, and Berry Global.

While our Language work package is certainly distinct, it is also designed to feed into and simultaneously be informed by the other areas of research on the MHR project. Our geographer colleagues in the Change team, for instance, are documenting the everyday lives of members of the public by observing and photographing their engagement with plastics and reuse within the home. Meanwhile, psychologists on the Willingness team are running controlled experiments to determine the limits of perceived aging and staining that British consumers are likely to accept in reusable plastic food containers (Baird et al. 2022). Elsewhere in the project, mechanical engineers are submitting candidate container materials to a range of stress tests, such as repeated scratching and industrial washing cycles, while chemical engineers are calculating the most ecologically viable packaging solutions by means of detailed life-cycle assessments. Our ecolinguistic work has closer ties with some of these work packages than with others, but ultimately learns from, and can contribute to, all areas of this multidisciplinary research.

The cross-disciplinary relevance of (eco)linguistics is not always obvious to all stakeholders, however. Of the ten university-led projects participating in this UKRI challenge, MHR is the only one to include a work package dedicated specifically to language. Reasonably, one might ask where linguists, with little to no background in plastics research, fit when it comes to research on packaging technology and polymer science. We would argue that our limited background in plastics is not only acceptable, for linguistic analysis, but in fact preferable. To be new to a subject can be an advantage for linguists, according to Stibbe, as “the stories that a discipline are based on may be so ingrained within the discipline that they go unnoticed” (Stibbe 2021a: 80). Drawing on Meyer and Land’s (2005) work on “threshold concepts” – the concepts that, once understood, irreversibly change one’s outlook and can even lead to shifts in disciplinary identity – Stibbe proposes that it may be better for discourses to be analyzed by those who have not yet crossed such thresholds (Stibbe 2021a: 80). Another central argument in his discussion is that ecolinguistics is not a subject or a methodology but can be considered a *transdisciplinary movement* that is not to be confined to the linguistic; to do ecolinguistic work is to be engaged in the “real world” and to have an “ethical and practical dimension” (Stibbe 2021a: 84). It makes sense, as we see it, to try to use (eco)linguistic research to transcend the disciplinary boundaries in a project as broad and complex as this one, and we recognize the general lack of linguistics in plastic research as a significant disciplinary gap.

## 3 Methodology

The aims of our research on *Many Happy Returns* are as follows: (i) to understand how people think about plastic (re)use by examining their language; (ii) to assess how well public perceptions appear to align with public-facing information on reuse; and (iii) to inform best-practice recommendations for communication around plastic reuse.

Given the usefulness of corpus linguistics as a means of analyzing large amounts of language data, we opted to build a corpus of relevant language and then analyze it using corpus software. This is (only ever) the first step, however, as all corpus output needs to be interpreted using the analyst’s chosen research methods and through the lens of their research philosophy. In this section, we describe our ecological philosophy for this research followed by the means by which we gathered and analyzed our corpus data.

### 3.1 Ecosophy

Over the course of our research on this project, we developed and subsequently formalized our ecosophy, or ecological philosophy. Now commonplace in ecological and ecolinguistic research, the ecosophy is an ethical framework to be set out at the start of the work, inspired principally by Naess (1990). It provides a philosophical grounding for the research and an ethical standard against which we can compare instances of language to determine whether or not they align with our values and overall mission (the overarching mission of our wider project being a reduction in single-use plastics). Drawing on Guattari’s (2000) concept of ecosophy, we emphasize the need for an integrated understanding of ecology, i.e. one that considers the interconnectedness of social, mental, and environmental ecologies. Following Stibbe (2021b) and others working in ecolinguistics, we present the main tenets of our ecosophy (E) as a list of values: (E1) Protection (of the planet and its inhabitants over profits); (E2) Recognizing the rights of all (to health, to safety, to wellbeing, to a future); (E3) Systems thinking (i.e. recognizing a network of causality, rather than placing full blame or onus on individual actors); and (E4) Fairness (towards all actors in the network). These values do not preclude the conventional ecosophy tenets (e.g. wellbeing, social justice, care, and compassion for others); rather, we have chosen to foreground these priorities to meet the needs of our research, which is especially social, political, and industrial in focus. In order to achieve ecological wellbeing for our planet as a whole, we must first, in this instance, envisage ways of addressing the material problems of logistics and infrastructure while reimagining the rights and responsibilities of each party involved.

It should be clarified here, for those unfamiliar with ecosophies, that while they are expressions of one’s values, they are also based in evidence and are subject to change as new evidence comes to light (Stibbe 2021b). As briefly described at the outset of this article, there is currently significant evidence to suggest that the production, consumption, and disposal of plastics is a major source of harm to our planet and its inhabitants (Rochman et al. 2013), whether in the form of carbon emissions from petrochemical industries (Zheng and Suh 2019) and the social injustices this pollution creates (UNEP 2021); plastic’s implications for the climate (Shen et al. 2020; Stoett and Vince 2019); plastic waste overwhelming communities around the world (Letcher 2020), constituting, some have argued, colonialism (Liboiron 2021); or harmful chemicals and microplastics entering ecosystems (Rochman et al. 2016; Verma et al. 2016), nonhuman bodies (Haave et al. 2021) and our bodies (Ragusa et al. 2021), the consequences of which are still relatively poorly understood (Nava and Leoni 2021; Vethaak and Legler 2021). These harms and hazards are to be weighed against plastic’s benefits, such as its convenience and affordability; its light weight for transport; its vast contributions to food provision, safety, hygiene, and medical care; and its central role as a flexible construction material in everyday artifacts such as technology and clothing (Andrady and Neal 2009; George 2020). With all of these factors in mind, we have designed our ecosophy around the values that we deem to be logically and morally relevant for the wellbeing of our planet as a whole when considering the supply and use of plastics, particularly single-use plastics.

### 3.2 Data

Given that one of our main objectives on the MHR project is to compare the language of plastics producers and plastics professionals with the language of members of the public, i.e. non-specialists, we set about building a corpus with two parts, each of which can also be considered a corpus in its own right. Corpus 1 is comprised of plastic- and reuse-oriented language of governmental and public-facing bodies, while Corpus 2 features language of members of the public around those same themes. Together, they form the MHR Corpus.

For Corpus 1, we identified two main sources of online, publicly available text reflecting governmental and public-facing plastics-oriented discourse: webpages from UK campaigns on recycling and plastic information (namely ‘Recycle Now’ and ‘Clear on Plastics’); and guidance from the UK government on refuse disposal across different local authority areas (‘Gov.uk’ council webpages). For Recycle Now and Clear on Plastics, the websites were small enough that it was possible to manually identify the sections of the websites deemed relevant to this work. The URLs for these pages were then fed into a freely available corpus-building program, BootCat,8
https://bootcat.dipintra.it/ (accessed 6 May 2021). which scraped the text from the webpages and exported it to files that could then be cleaned, tagged, and added to the Corpus 1 collection. BootCat is typically used to build a corpus from scratch, i.e. by specifying key terms of interest (‘seeds’) which it then combines randomly into search queries (‘tuples’) to be used in Google searches to generate the relevant URLs, and this is how we used BootCat to collect the data from the ‘Gov.uk’ webpages (see Appendix A for the list of tuples used).

Another major source of public-facing discourse around plastics, especially packaging, is that of UK supermarket websites. Using BootCat again, we identified and scraped relevant webpages from the websites of eight of the UK’s largest supermarkets, with varying degrees of success: Co-op, Ocado, M&S, Asda, Sainsbury’s, Morrisons, Tesco, and Waitrose. The tuples used to gather this data are listed in Appendix A.

Finally, to hear directly from those who work with plastic packaging in a professional capacity – whether in terms of labeling, sales, design, or campaigning – we disseminated an industry survey posing questions specifically around language and labeling practices for plastic packaging (see Appendix A for the list of questions). We received 28 responses, with respondents ranging from retailer and manufacturer representatives to business owners, packaging designers, and consultants, among others. The responses to the survey, though relatively small in size, were included in Corpus 1 along with the public guidance and the supermarket website texts. Table 1 shows the final composition of Corpus 1.

**Table 1:** Corpus 1 composition.

Corpus 1: public-facing discourse				
Subcorpus	Component	Files	Tokens	Total tokens
Public/gov. guidance	Recycle now	144	29,369	365,855
	Clear on plastics	17	9,109	
	Gov.uk webpages	336	327,377	
Supermarket website texts	Co-op	23	8,606	1,083,209
	Ocado	274	66,820	
	M&S	136	162,254	
	Asda	83	59,134	
	Sainsbury’s	51	307,101	
	Morrisons	22	18,944	
	Tesco	134	428,593	
	Waitrose	98	31,757	
Industry language survey	Survey responses	28	4,413	4,413
**Corpus 1 totals**		**1,346**		**1,453,477**

Next, in order to build up a broad picture of how members of the general British public speak and write about plastics, packaging, and reuse, we gathered linguistic data from a range of sources for inclusion in Corpus 2. Twitter was identified as an abundant source of geolocated statements from members of the public, and using the Twitter Developer Academic Research product track, we downloaded 66,393 tweets via the Twitter API.9Using the ‘searchtweets-v2’ Python package, available at https://pypi.org/project/searchtweets-v2/. The criteria for tweets to be downloaded were: (i) they must contain one or more of the relevant terms (see Appendix B); (ii) they must be in English and be geotagged to the UK, to try and capture British English language patterns; (iii) they must fall within the timeframe January 2016 to January 2021; and (iv) they must not be a retweet, so as to keep the level of corpus noise to a minimum.

We then selected two general-discussion internet forums which have a large UK userbase and identified relevant subforums and threads within them: the social media platform Reddit, and the UK-oriented parenting forum Mumsnet. Within Reddit, we located three suitable subreddits (‘AskUK’, ‘CasualUK’, and ‘BritishProblems’) and then used the Reddit API10Via Joseph Lai’s ‘Universal Reddit Scraper’ Python package, available at https://github.com/JosephLai241/URS. to download threads from within those subreddits that met our search term criteria (see Appendix B for these terms), posted between January 2016 and March 2021. For Mumsnet, given that there is no API available and the website is more difficult to scrape, we used a combination of BootCat and Octoparse,11
https://www.octoparse.com/ (accessed 6 May 2021). another freely available webpage scraper, to locate and download relevant threads that were posted within the same timeframe as those of Reddit. The BootCat tuples used to determine the Mumsnet threads are given in Appendix B. In all cases of social media and forum post scraping, only publicly available posts and comments were gathered for inclusion in the corpus, and all usernames were pseudonymized.

In addition to the social media and forum data that we downloaded, we also held online focus group discussions with members of the British public to explore views on plastics and packaging and to generate spoken language data to be included in the corpus. We ran five focus groups in total, involving 28 participants: three of the focus groups were recruited using participants from a previous experiment run by our psychologist colleagues on the MHR project, who had consented to being contacted about future experiments; and the other two were recruited using a combination of online calls for participation via environmentally oriented, UK-based Facebook groups (e.g. ‘Zero-Waste London’, ‘Sustainable Lifestyle UK’) and a call via the mailing list of one of our environmental charity project partners, City to Sea. As such, the first three focus groups involved members of the public who do not explicitly identify as being pro-environmental, and the final two involved those who do. The transcripts of the five focus group recordings were added to the social media and forum data to complete Corpus 2. Table 2 shows the final Corpus 2 composition.

**Table 2:** Corpus 2 composition.

Corpus 2: consumer-generated discourse				
Subcorpus	Component	Files	Tokens	Total tokens
Social media and forum posts	Twitter	2	1,736,578	4,096,959
	Reddit	399	490,297	
	Mumsnet	358	1,870,084	
Focus groups	Focus group 1	1	11,196	59,148
	Focus group 2	1	11,239	
	Focus group 3	1	10,854	
	Focus group 4	1	13,647	
	Focus group 5	1	12,212	
**Corpus 2 totals**		**864**		**4,156,107**

We note here that there can be some contention around the inclusion of elicited language data in a corpus. We bear in mind for our analysis that this kind of data (focus groups, interviews, surveys, etc.) is of a slightly different nature to naturally occurring language data, but we are also not laboring under any illusions that an “authentic language” corpus is somehow insulated from bias, sampling issues, and so on. We also highlight here that there is a distinction to be drawn between written and spoken language data; the focus group transcripts are an example of the latter.

Both Corpus 1 and 2 – which, put together, form the MHR Corpus – will be made available to download, open-access, along with the full corpus documentation.12Please contact the authors for more information on the release of the corpus.


### 3.3 Analysis

Both corpora, Corpus 1 and 2, were loaded into AntConc,13
https://www.laurenceanthony.net/software/antconc/ (accessed 6 May 2021). a freely available corpus analysis program. Standard initial corpus analyses were carried out for each corpus: the generation of a wordlist (a list of all of the words, or ‘types’, in the corpus, in descending order of frequency); a keywords analysis (a comparison of the wordlists of each of the corpora to show which terms are unusually (in)frequent, or statistically ‘key’); and a clusters analysis, which is similar to a wordlist but it lists the most frequent phrases, or ‘n-grams’, rather than single words. Clusters can be requested with certain parameters, e.g. by asking the program to only return n-grams between two and three words long, for instance, or to retrieve clusters that contain a term of interest.

To run a keywords analysis, it is necessary to specify a reference corpus against which the target corpus (the corpus of interest) can be compared. As one of our research aims is to assess in what ways the language of public-facing bodies (Corpus 1) and members of the public (Corpus 2) differ, our two MHR corpora were each used as a reference corpus for the other. The rationale for this is that they are both built around the same themes (plastic, packaging, reuse, and recycling) and by comparing their wordlists, we are able to establish which words and phrases are statistically more salient than expected, considering that both corpora are already based on the same themes (if we were to take, for example, a general English corpus as a reference corpus, as it would most likely simply bring up the predicted themes of plastic, packaging, reuse, and recycling). For Corpus 2, we decided to also run a second, heuristic keywords analysis against a different reference corpus to see if we could account for genre effects on pronoun use. This second reference corpus was an ad hoc collection of similar texts (social media posts, website comments, focus group transcripts) from previous corpus-building projects, unfortunately not shareable for public use. Appendix C gives more information about the texts in this ad hoc corpus.

Running the above analyses produced simple and typical corpus software output, i.e. lists of terms and frequencies (examples of which are given in Section 4), but this output alone is not sufficient for a full, critical, (eco)linguistic analysis. These raw outputs were therefore submitted to further, manual analyses, including annotation of samples of concordance lines (keywords shown in their contexts), further corpus querying of terms of interest as and when they arose, and finally a critical evaluation of these findings from ecolinguistic and cognitive linguistic perspectives, also taking into account the findings in the literature.

While corpus methods go some way towards reducing researcher bias in linguistic research, and this is one of its strengths – it draws on very large amounts of data which enables more reliable generalizations, and relies on machines to identify salient terms for closer investigation – it is not entirely insulated from bias. Baker (2015), for instance, demonstrated that when several corpus linguists are given the same corpus data and the same research question, there is no guarantee that they will reach the same conclusions in a discourse analysis task. In this respect, the application of our ecosophy was of central relevance to our study and to our overall findings, as illustrated in the following section.

## 4 Findings

### 4.1 Corpus outputs

The wordlists for both corpora are given, truncated to the top 50, in Appendix D. To summarize some of the key findings from the basic corpus analyses described in the section above, we can report that:

– There is a far higher preoccupation with plastic, generally, in Corpus 2 (social media texts and focus groups) than there is in Corpus 1 (public-facing texts and the industry survey); plastic* occurred 16,030 times per million words (pmw) in Corpus 2, compared with 5,597 times pmw in Corpus 1.
– According to the lists of keywords (shown in Table 3), *our* is the most statistically salient14Keyness scores for keywords were calculated in AntConc using log-likelihood (4-term), *p* < 0.05 (Bonferroni-adjusted). term in Corpus 1, while in Corpus 2 it’s *I* (or *plastic*, if comparing against the ad hoc reference corpus).
– The keyword lists reflect the more corporate nature of Corpus 1 (e.g. *financial*, *customers*, *report*, *business*, *colleagues*, *executive*, *cash*, *value*, *assets*), as well as the interpersonal (*I*, *my*, *you*, *me*, *they*) and conflicted (*but*, *just*, *don[’t]*, *think*) sentiments of Corpus 2. When compared against the ad hoc reference corpus, which is of a similar (mainly social media) genre to Corpus 2, we can also see confirmation of the plastic- and packaging-oriented nature of Corpus 2, along with some statistically salient pronouns (*we*, *they*, *them*).
– At a glance, we can see that there are marked lexical differences in the ways retailers (Corpus 1) and members of the British public (Corpus 2) communicate about plastics and reuse.

**Table 3:** Keywords for Corpus 1 and 2.

	Corpus 1 (vs. Corpus 2)		Corpus 2 (vs. Corpus 1)		Corpus 2 (vs. ad hoc ref. corpus)	
	Keyword	Keyness	Keyword	Keyness	Keyword	Keyness
**1**	*our*	12,636.42	*I*	47,939.88	*plastic*	22,419.81
**2**	*financial*	9,153.85	*plastic*	13,166.00	*packaging*	5,613.13
**3**	*group*	8,538.49	*it*	13,129.13	*to*	4,969.56
**4**	*customers*	7,069.67	*t*	10,638.83	*use*	4,606.01
**5**	*and*	5,029.64	*my*	10,425.65	*waste*	4,347.54
**6**	*report*	4,791.03	*but*	7,573.61	*we*	4,080.50
**7**	*tesco*	4,718.68	*just*	6,257.93	*bottles*	3,567.50
**8**	*business*	4,675.67	*so*	5,605.76	*they*	3,012.18
**9**	*colleagues*	3,959.56	*you*	5,511.55	*bags*	2,973.84
**10**	*plc*	3,836.81	*me*	4,709.53	*the*	2,961.63
**11**	*of*	3,835.29	*don*	4,054.08	*recycling*	2,690.42
**12**	*by*	3,825.75	*think*	3,388.11	*single*	2,400.53
**13**	*net*	3,584.17	*they*	3,368.89	*plastics*	2,174.48
**14**	*executive*	3,579.68	*get*	3,199.52	*recycled*	1,769.51
**15**	*cash*	3,564.40	*a*	3,051.08	*reduce*	1,675.84
**16**	*value*	3,563.32	*would*	2,690.20	*paper*	1,668.94
**17**	*stores*	3,496.56	*buy*	2,631.77	*that*	1,629.36
**18**	*assets*	3,448.41	*like*	2,624.57	*them*	1,598.98
**19**	*committee*	3,408.45	*stuff*	2,617.85	*bottle*	1,582.13
**20**	*performance*	3,332.18	*really*	2,508.90	*recycle*	1,533.57

Results such as these are quantitatively substantiated, and – given the same corpus – reproducible by other researchers. They also provide us with a defensible order in which to prioritize terms of interest for closer investigation; higher frequencies of terms indicate a greater preoccupation with those terms and their associated concepts (Schmid 2007: 119–120). However, meaningful interpretation of corpus data always requires careful and critical analysis through chosen analytical frameworks; in our case, we chose to consider the corpus outputs through the lens of cognitive linguistics and with our predefined ecosophy in mind. Our findings are reported in more detail elsewhere, particularly in relation to metaphor and narrative (forthcoming), and for the purposes of this article we will discuss just a few short examples as a demonstration of a corpus-ecolinguistic analysis.

### 4.2 Applying the ecosophy: Key pronouns

Looking at Table 3, we see that a few personal pronouns ranked especially high in our keywords analysis results, notably *our* in Corpus 1, and *I* in Corpus 2. Here, we take these two terms as starting points for a discussion around the role of an ecosophy in corpus linguistic analysis. For the purposes of this article, which we dedicate to the demonstration of an ecosophy in practice for the development of communications recommendations, we do not go into extensive depth on each result but rather seek to relate these results to our ecosophy.

#### 4.2.1 Corpus 1: *our* (and *our customers*)

Beginning with *our*, the highest-ranking keyword for Corpus 1, we found that its most frequently occurring collocate, or neighboring word, is, by a long way, *customers* (the phrase *our customers* occurs 1,361 times in Corpus 1; the single word *customers* occurs 4,052 times), followed by (*our*)* business* (*n* = 509), (*our*)* own* (*n* = 462) and (*our*)* stores* (*n* = 449). Given the high frequency of *customers* and *our customers*, we decided to take *customers* as a starting point for closer investigation.

A random sample of 200 concordance lines of *customers* was exported from AntConc to Excel and annotated for thematic roles (see Hilpert [2014: 27] for a list and discussion of frequently used thematic/semantic roles). We found that in half (*n* = 100) of all cases, the *customers* were construed solely as either Beneficiaries or Recipients, while 72 lines featured *customers* in the role of Agent, with 30 of these *customers* situated as Agent only by virtue of being simultaneously a Beneficiary, Recipient, or Theme. Some examples of these are given below in (1)–(4).

(1)

**Table:** 

The positive impact of this will be far reaching: by helping our **customers** eat more sustainable diets, restoring nature in food production and eliminating waste from the retail industry. [Beneficiary, Agent]

(2)

**Table:** 

We were the first UK retailer to remove multi-buys on food products from our shelves – helping **customers** avoid waste at home and enabling us to forecast demand better. [Beneficiary, Agent]

(3)

**Table:** 

[…] we are now extending Plan A further by encouraging all of our **customers** and employees to live ‘greener lifestyles’ […]. [Theme, Agent]

(4)

**Table:** 

The pop-up store in London was a store with a difference, giving **customers** the opportunity to shop for others. [Recipient, Agent]

In these types of cases, *customers* are being enabled by companies, i.e. stores, to make more ecological or ethical choices. Where *customers* are presented solely as Agents, we see examples such as (5)–(8).

(5)

**Table:** 

Every three months **customers** can vote with a blue token for the project that they would like to receive funding in their local community. [Agent]

(6)

**Table:** 

**Customers** can donate clothing, shoes and textiles that are from any brand, and of any quality, in our conveniently located collection units at the front of store. [Agent]

(7)

**Table:** 

Asda **customers** have still been able to support the charity by donating their unwanted items to the new ‘drop and shop’ donation point. [Agent]

(8)

**Table:** 

From today, **customers** can deposit plastic bottles of any size up to 3 L and aluminium drinks cans in a machine at the store entrance, in exchange for a coupon that’s worth 5p per item towards their shopping. [Agent]

Notably, we see here how *customers* are still implicitly construed as Beneficiaries and Recipients, either through the use of potentials (*can*, *have been able to*) or through the positioning of the store and its facilities as the enabling actor in these constructions (*in our conveniently located collection units at the front of the store*, *to the new ‘drop and shop’ donation point*, *in a machine at the store entrance*). These examples also represent another trend in the customers-as-Agents data, which is that the active processes they are engaging in are often related to charity (e.g. *donate*, *support*, *vote for*) or consumption (e.g. *buy*, *purchase*, *shop*).

The overall depiction of *customers*, and particularly *our customers*, is that of the customer as a kind of dependent and the company as a kind of enabler. *Customers*, even in cases when they are technically described as agentic, are portrayed as achieving something beneficial for the environment or for the community only by virtue of the company’s providing the opportunity and means to “their” (*our*) customers. Effective actions against ecological problems are, for *customers*, the acts of donating goods, purchasing goods, or appealing to the company to take particular courses of action on their behalf. Rather than being positioned as leaders or capable agents in themselves, (*our*) *customers* are typically being *helped* or *encouraged* to make responsible choices, or in other cases *supported* and *protected*, reinforcing the imbalances of power and responsibility between customer and company, similar to that of a child and their parent or caregiver. We present a more detailed cognitive linguistic exploration of this corporation is a parent metaphor elsewhere (forthcoming), but for the purposes of this discussion about ecosophy-assisted corpus linguistics, we hope that this level of detail will suffice.

Returning to our ecosophy, then, we ask ourselves: (E1) Does this type of discourse promote protection (of the planet and its inhabitants over profits)? (E2) Does it recognize the rights of all? (E3) Does it engage with systems thinking? (E4) Does it promote fairness? Reflecting on these values leads us into a deeper and more critical evaluation of the language patterns being uncovered: is there something (un)helpful or (un)ecological about a parent–child metaphor when it comes to discussions of plastics and packaging, for instance? And if so, what is it, exactly? In response to the tenets of our ecosophy, we might comment that (E1) protection of the planet and its inhabitants *is* being promoted here, but only to the extent that it can coexist alongside protection of the customer and their shopping experience, as well as the companies and their “green” credentials, and not to the extent that the protection of the planet takes priority over the company’s profits; (E2) the rights of customers to health, safety, well-being, and a future are framed as provisions offered by a company in the form of goods and services, rather than via customer agency, autonomy, or independence; (E3) systems thinking (recognizing a network of causality) is presented here in a rather skewed sense, with customers represented as needing to reduce their individual waste first and foremost, the companies simply coming to their aid in this; and (E4) with all of these points in mind, fairness is called into question: while companies’ efforts to make “greener” choices are recognized as positive steps, companies are also positioning themselves as the key sources of power and protection, while presenting customers as the responsible parties to be served and supported in their consumerism.

#### 4.2.2 Corpus 2: *I*

As shown in Table 3, *I* was the top keyword for Corpus 2 when comparing frequencies against those of Corpus 1. We suspected that this result may be due to genre differences (first-person pronouns like *I* and *me* are typical in social media, and less typical in public-facing, corporate texts) and so we ran a second keywords analysis against an ad hoc reference corpus comprising texts from more similar genres (social media posts, website comments, focus group transcripts, etc.). This second analysis confirmed that while *I* is statistically salient in Corpus 2 when we compare it against Corpus 1, *I* does not feature in the top 20 keywords when compared against the corpus of texts from similar genres, suggesting that *I* is indeed a genre feature. When compared against the ad hoc corpus, the statistically key pronouns were found instead to be *we* (ranked #6), *they* (#8), and *them* (#18). Nonetheless, *I* is still a distinctive marker of our corpus of the language of members of the public concerning plastics, packaging, and reuse; it is the third most frequent word overall in Corpus 2 (see the wordlist in Appendix D), occurring more frequently even than words such as *a*, *and*, and *of*. For this reason, and for the purposes of demonstrating the application of our ecosophy in a limited space, we will discuss here just the pronoun *I* in Corpus 2. We discuss *we*, *they*, and *them* in a separate article, forthcoming.

To investigate this very high frequency of *I* in Corpus 2, we used AntConc to generate a list of the most frequently occurring *I* phrases in the Corpus, given in Table 4.15AntConc tokenizes contracted words (e.g. *I’m*, *don’t*) as two tokens, hence why most of these 4-grams are three words long.


**Table 4:** Most frequently occurring 4-gram, right-hand-side clusters of *I* in Corpus 2.

	Top *I*-initial 4-grams in Corpus 2		
	Frequency (raw)	Range	Cluster
**1**	737	4	*I’ve just signed*
**2**	640	221	*I don’t think*
**3**	532	204	*I don’t know*
**4**	517	144	*I’m going to*
**5**	402	163	*I don’t have*
**6**	329	143	*I’m not sure*
**7**	321	134	*I think it’s*
**8**	306	116	*I don’t want*
**9**	227	79	*I’m trying to*
**10**	196	2	*I demand urgent government*

Note: “Range” refers to the number of files in which the clusters appear.

From a cursory glance, we can see that there are two phrases that, unlike the others, are localized to just a few files in the corpus: *I’ve just signed* (ranked #1) and *I demand urgent government* (#10). These are both examples of petition text that has been copied and shared verbatim on Twitter (all Tweets are spread across just two files in the corpus, hence the low range for these particular results). We also note that five out of the top ten clusters are negated phrases (e.g. *I don’t think*, *I don’t know*, *I don’t want*). Although just a snapshot of the “I” instances in Corpus 2, Table 4 paints the picture of a frustrated, uncertain, and disempowered community of citizens faced with the global problem of plastic waste. The two petition examples mentioned above – *I’ve just signed* and *I demand urgent government*, shown in Examples (9) and (10) – speak to the theme of reliance on governing or corporate bodies that we saw in the *our customers* examples of Corpus 1.

(9)

**Table:** 

**I’ve just signed** a petition calling on @amazon to change their packaging to plastic-free options that can be recycled easily after use [URL] via @38_degrees.

(10)

**Table:** 

**I demand urgent government** action to reduce manufacturers’ and retailers’ use of plastics. Join me and sign the @friends_earth petition: [URL].

These petition cases are less phraseologically relevant to our study than the other clusters in Table 4 in that they are essentially copied-and-pasted duplicates, but their frequency in our corpus of Tweets nonetheless indicates a tendency to appeal to “parent” entities for action on plastics.

Evidently, there is some degree of effort – and struggle – on the part of these language users in Corpus 2 to not only make a difference but to try and make sense of the situation as a whole. Taking a closer look at *I don’t think* (ranked #2) and *I think it’s* (#7), we find a combination of pessimistic statements around plastics and the environment (see Examples 11–13) and attempts to understand the nature of this very complex issue (Examples 14 and 15). There is also a clear sense of skepticism about what might constitute effective solutions to the problem, and about the willingness of others to make ecological choices (Examples 16 and 17).

(11)

**Table:** 

**I don’t think** there’s much I can meaningfully do on an individual level.

(12)

**Table:** 

**I think it’s** too little too late.

(13)

**Table:** 

**I don’t think** governments are prepared to make that investment, plus the storage of waste plastic is a problem, who wants it on their doorstep, it’s unsightly and smells. No easy solution unfortunately.

(14)

**Table:** 

**I think it’s** entitlement and selfishness too.

(15)

**Table:** 

**I don’t think** it’s as simple as people not caring but feeling impotent in the face of a global situation that is very hard, at times, to be tangible for the masses.

(16)

**Table:** 

**I don’t think** a Keep Britain Tidy campaign would work nowadays – not enough national identity.

(17)

**Table:** 

**I don’t think** education is the answer.

A similar sentiment is found in the instances of *I don’t know* (ranked #3) and *I’m not sure* (#6). Language users appear to be tentatively proposing solutions to the environmental problems posed by plastics, but are highly uncertain (Examples 18–20), while also expressing sentiments of skepticism and hopelessness (Examples 21 and 22).

(18)

**Table:** 

Isn’t washing powder in a cardboard box the best option? **I don’t know**, maybe it isn’t?

(19)

**Table:** 

They might be made from recycled material but **I’m not sure** they are recyclable or biodegradable.

(20)

**Table:** 

**I don’t know**. The answer isn’t simple.

(21)

**Table:** 

**I don’t know** how this can be tackled without a massive move towards being a less consumerist society but that doesn’t seem popular.

(22)

**Table:** 

I’d like to refuse to attend training where single use plastic is used but **I’m not sure** it’s possible.

*I’m going to* (ranked #4) and *I’m trying to* (#9) raise the themes of effort, intentions, and aspirations, often involving a struggle or frustration, especially in the case of *I’m trying to* (Examples 23–26). In combination with *I don’t have* (#5) and *I don’t want* (#8), we receive the overall impression that consumers of plastic do not feel adequately equipped to live as ecologically as they would like (Examples 27 and 28), or find the prospect of the more ecological options available to them unattractive or unreasonable (Examples 29 and 30).

(23)

**Table:** 

**I’m going to** start using bamboo toothbrushes and bar soap too.

(24)

**Table:** 

**I’m going to** aim to go completely plastic bottle free.

(25)

**Table:** 

**I’m trying to** be zero waste and plastic free, it’s difficult because literally everything is wrapped in plastic or designed to break it seems.

(26)

**Table:** 

**I’m trying to** buy wooden toys only now, but that’s a challenge.

(27)

**Table:** 

**I don’t have** the time to shop at markets which is what I guess we should be doing.

(28)

**Table:** 

I agree on the supermarket packaging. Sadly, **I don’t have** a greengrocer or decent market nearby.

(29)

**Table:** 

**I don’t want** to be scraping out the contents of the bathroom bin.

(30)

**Table:** 

**I don’t want** to have to pay more for the same veg or fruit because I chose to reduce my plastic.

Returning to our ecosophy and how we can employ it in the appraisal of these corpus examples, we might comment that: (E1) with respect to protection of the planet and its inhabitants, consumers evidently do not feel adequately protected from the harmful consequences of plastic use, and although they would often aim to try to protect the planet and its inhabitants, they do not feel capable of being effective protectors themselves; (E2) the rights of all inhabitants of the planet to health, safety, wellbeing, and a future are not being foregrounded in this discourse, and instead consumers appear to be struggling with, and ultimately resigning themselves to an undesirable situation in which the negative impact of plastics is being felt by all; (E3) systems thinking is not being employed here in a particularly salient way, as the onus of planet protection is generally being attributed, or rather relinquished, to the “parent” bodies in positions of capitalistic power. The proportionate power and responsibility of members of the public within this network of actors are not acknowledged here particularly strongly, and instead there is a clear reliance on institutions to take effective action; (E4) these examples depict a generally unfair state of affairs in which the planet and its citizens are suffering from the effects of plastic’s enmeshment in our society and environments, and are unable to see a feasible way out. Interestingly, some of this language also suggests a reluctance on the part of individuals to engage in inconvenient or unappealing behaviors that would potentially stand to benefit the planet as a whole. Whether this is unfair of those individuals toward other actors in the network, or whether it is unfair for consumers to be put in this position, is open to discussion.

## 5 Discussion and recommendations

In our analysis, we have focused on just a couple of corpus experiment results related to the language of plastics to demonstrate a possible application of ecosophy to a discourse analysis task. Rather than focusing specifically on the language of reuse, we have shown that the outputs of basic, bottom-up, and data-driven corpus analysis measures can easily be plugged into an ecosophy and vice versa. The corpus was built using specific seed terms related to plastics and reuse, so by taking a bottom-up approach, we expect to see findings around these general topics. We might also have searched, in a top-down way, for terms such as ‘reuse’, ‘return’, ‘refill’, and ‘repurpose’ to locate explicit mentions of plastic reuse, but in this particular study we have opted to let the frequency and statistical salience of corpus terms speak for themselves. Themes of reuse have instead emerged in more subtle ways: citizens reported feeling impotent and frustrated about their role in reducing single-use plastics; there was a persistent sense, from both corpora, that individuals cannot make a difference and instead must rely on the actions of corporations; citizens reported feelings of confusion about whether a course of action is ecologically beneficial or not; and there was evidently a sense of reluctance and even disgust associated with some of the actions involved in circular packaging (*I don’t want to be scraping out the contents of the bathroom bin*).

The purpose of using an ecosophy to evaluate examples of language is to determine which narratives are aligned with the ecosophy and are therefore ecologically beneficial, and which ones are in conflict with the ecosophy and therefore considered harmful. Based on what we have so far observed in the findings discussed in this article, and also taking into account the findings reported in the literature, we propose the following preliminary recommendations (Rs) for communications around plastics and reuse.

– R1: An ecosophy should be explicitly drawn up and its tenets strongly foregrounded in proposed messaging strategies. In the case of our ecosophy and for our communicative purposes, this could lead to constructions such as: *Let’s all work together to protect our environment* [protection, systems thinking]; *Being fair means securing a future for everyone* [fairness, rights]; *Thank you for thinking of others and bringing your reusable container* [fairness, protection]; *We all have a role to play in making sure plastics don’t reach our oceans* [systems thinking, protection].
– R2: In line with previous findings on framing and polarity, and keeping in mind the largely negative valence of consumer language related to plastics and ecological practices (see Corpus 2 *I* phrases), positively and negatively framed messages should be used mindfully. Positive messages are preferred for high-level, abstract purposes, with negatively framed messages kept for low-level, concrete actions. In the case of promoting reusable plastic packaging systems, this translates to a positive framing of the phenomenon of reuse, combined with a negative framing of concrete (non-)reuse actions, e.g. *Reuse is the new recycling; don’t get caught out without your container!*
– R3: Messaging strategy should be data-driven and tailored to the language of its target audience. In this study, we have identified discursive patterns indicating that both companies and consumers perceive institutions to be holding the key authority and power to make change in plastic packaging systems. This coincides with a narrative of impotence and hopelessness on the part of consumers. A tailored counter-narrative for a pro-reuse campaign might involve positive stories that subvert this view, e.g. *Corporations need your permission to create plastic waste; why give it to them?* or *Don’t wait for companies to act on plastics – you hold the power to make that change.* When it comes to responsible brand management and in-store messaging, companies might opt to disrupt the corporation is a parent metaphor by emphasizing the customer’s agency and adulthood instead. This might sound like: *Thank you for enabling us to do better*, or *Should we switch to reuse? We’re waiting on you to give us the signal.*
– R4: As supported by much of the literature, pro-environmental messaging should not be too forceful or assertive, unless it is safe to assume that the reader/hearer has already accepted the urgency of the problem. Our Corpus 1 analysis of *our* (*customers*) demonstrated that customers are currently used to being approached very gently with regard to their environmental responsibilities. To achieve our aims with circular packaging systems, we should be reminding citizens of reasons and opportunities to engage in reuse and encouraging their participation without promoting it too repetitively or aggressively.
– R5: Narratives and stories, e.g. of empowerment, fairness, rights, and protection, should be prioritized over attempts to merely educate or convey “facts”. Facts are not nearly as salient in the minds of hearers and readers as stories, and we must first establish the stories being perpetuated in the public sphere before devising counter-stories that fit these mental models. Public “education” campaigns should therefore be centered around a prototypical story involving, e.g. a hero, an obstacle, and a path to victory. Such themes, or text worlds, can be encoded in relatively little text.

Methodologically, we also recommend that multidisciplinary research teams consciously engage with, and establish, a collective ecosophy from the beginning of a project. Not only will this help to produce a cohesive research philosophy and mission statement across all of the subteams, regardless of the subject matter, but it will also ensure that any linguistic recommendations can be tailored effectively to the goals of the research. In the case of our project, the ecosophy remained limited to the linguistics work package. Its usefulness in framing both our methodological and analytical approaches would lead us to propose extending it across all strands of a multidisciplinary project in future collaborations.

## 6 Conclusions

This article has presented some findings around the language of plastics, packaging, and reuse through an ecolinguistic lens, drawing on methods and insights from corpus linguistics and cognitive linguistics. Utilizing a thematic corpus of 5.6 million words, we located key terms and phrases relevant to our research questions around consumer attitudes towards plastic (re)use as well as how these sentiments align with those of public-facing sources such as supermarket and governmental websites. Analysis of these key terms demonstrated that there are recurring narratives in both consumer-generated and public-facing discourses that are in conflict with the tenets of our pre-defined ecosophy, and as such are deemed potentially harmful or hindering. In response, we have proposed some preliminary recommendations for effective communications that foreground the tenets of our ecosophy and aim to foster a positive view of reuse. Our analysis also suggests that in order to make meaningful changes in packaging reuse behaviors, it is highly likely that deeply ingrained cultural stories around power, rights, and responsibilities will need to be directly challenged.

The explicit application of an ecosophy in this discourse analytic task has helped to define a standard of acceptability in environmental communication and has enabled us to recommend alternatives based on a transparent set of principles. Far from introducing more bias, the ecosophy has provided a clear framework of values against which we, and other researchers, can compare examples of language, thus improving reproducibility in (corpus-assisted) discourse analysis. It follows that implementing an ecosophy also stands to improve measurability of the “greenness” of communications, providing that the ecosophy itself is appropriately constructed around “green” priorities. We should add here that ecosophy can, and should, be adapted to the domain and goals of a discourse analysis task, whether around environmental topics or other subjects of social and moral importance; it is, essentially, an explicit statement of one’s research philosophy and relevant values. Ecosophy can also be applied to discourse of other modalities and not only text, which may be of use to researchers in business, marketing, and media studies.

The findings reported in this article provide empirical linguistic evidence for a state of affairs that perhaps already seems obvious or is taken for granted: that citizens feel heavily reliant on companies and other institutions to effect real change around plastics, packaging, and the environment. Using our ecosophy, we have been able to problematize this dynamic and to ask critical questions about whether or not this acceptance of power imbalance is necessarily conducive to a substantial change in packaging systems. We recognize here that there is an uncomfortable relationship between retailers and individuals due to forces of capitalism, and that for many people (and businesses) there simply is no other feasible option than the ones currently made available. We have also made reference in this article to the necessity and goodness of plastics in many scenarios for the survival and wellbeing of humans, and we do not seek to demonize plastics as a whole. Rather, we aim with this work to critically engage with the systems and networks that serve to perpetuate a way of being which is incompatible with our ecosophy and, indeed, with a viable future on this planet.

This study is part of ongoing research on the *Many Happy Returns* project, and we continue to investigate UK discourses around the themes of plastic packaging and reuse. We envisage that future work will entail closer examinations of salient stories and narratives, as well as more comprehensive guidelines on public-facing communications around plastics. We expect that this research will be of use not only academically but, crucially, in the development of effective science communications in the UK for circular plastic packaging systems.

## Appendices

### Appendix A: Corpus 1 construction details

**Gov.uk website tuples in BootCat:**

– *recycling plastic reuse*
– *recyclable reusable plastic*
– *plastic recycling reusable*
– *recycling reusable recyclable*
– *reuse recyclable plastic*
– *reusable reuse plastic*
– *reusable recyclable reuse*
– *reuse recycling recyclable*
– *plastic recyclable recycling*
– *reuse recycling reusable*

**Supermarket website tuples in BootCat:**

– *bag packaging reuse*
– *bag plastic recycle*
– *packaging plastic reuse*
– *plastic bag packaging*
– *recycle bag reuse*
– *recycle packaging bag*
– *recycle plastic packaging*
– *recycle reuse plastic*
– *reuse bag plastic*
– *reuse packaging recycle*

**Industry language survey questions:**

1. Do you or your organization have particular language policies or strategies when it comes to packaging, i.e. the text/pack copy?
2. Please elaborate on your answer.
3. In your view, or from the perspective of your organization, what makes a good packaging label? What information should be prioritized?
4. Can you describe your approach, or the approach of your organization, to supporting/point-of-sale information with regard to plastic packaging?
5. If you are involved in packaging and/or on-pack label design, are there any examples of labeling that you are especially proud of? Please tell us a bit about that. And if you aren’t involved in design, please tell us about some packaging/labeling that you especially like, and why.
6. What information would you wish to be included on packaging labels, if e.g. space and branding weren’t an issue?
7. If you are involved in packaging and/or label design, is there anything that you purposely avoid including on packaging labels?
8. What do you most want consumers to take away from the text on packaging labels? Is there any messaging that you feel isn’t landing with consumers?
9. Do you have any other thoughts or comments you would like to share?

### Appendix B: Corpus 2 construction details

**Twitter search term criteria:**

Mentions one or more of: *plastic*, *plastics*, *packaged*, *packaging.*

Also mentions one or more of: *food*, *sustainable*, *sustainably*, *waste*, *wastes*, *wasting*, *wasted*, *buy*, *buys*, *buying*, *bought*, “*single use”*, “*single-use”*, *disposable*, *disposables*, *dispose*, *disposed*, *disposes*, “*throwing away”*, “*throw away”*, “*thrown away”*, “*throws away”*, “*threw away”*, *throwaway*, *reduce*, *reducing*, *reduced*, *reduces*, *recycle*, *recycles*, *recycling*, *recycled*, *recyclable*, *reuse*, *reused*, *reuses*, *reusing*, *reusable*, *repurpose*, *repurposed*, *repurposing*, *zerowaste*, *zero-waste*, “*zero waste”*, *refill*, *refilled*, *refills*, *refilling*, *refillable*, *return*, *returned*, *returning*, *returns*, *returnable*, *bottle*, *bottles*, *container*, *containers*, *carton*, *cartons*, *tray*, *trays*, *lid*, *lids*, *wrap*, *wraps*, *wrapped*, *wrapping*, *packet*, *packets*, *bin*, *rubbish*, *trash*, *landfill*, *eco.*

**Reddit search term criteria:**

*plastic* AND (*packaging* OR *food* OR *sustainable* OR *“single use”* OR *single-use* OR *“disposable”* OR *“throw away”* OR *recycle* OR *recycling* OR *reuse* OR *zerowaste* OR *zero-waste* OR *“zero waste”* OR *refill* OR *return* OR *bottle* OR *container* OR *carton* OR *tray* OR *lid* OR *wrapped* OR *packet* OR *eco* OR *bin* OR *landfill*).

**Mumsnet tuples in BootCat:**

– *plastic packaging food*
– *plastic packaging sustainable*
– *plastic packaging sustainably*
– *plastic packaging waste*
– *plastic packaging buy*
– *plastic packaging “single use”*
– *plastic packaging single-use*
– *plastic packaging disposable*
– *plastic packaging “throwing away”*
– *plastic packaging “throw away”*
– *plastic packaging “thrown away”*
– *plastic packaging “throws away”*
– *plastic packaging “threw away”*
– *plastic packaging reduce*
– *plastic packaging recycle*
– *plastic packaging reuse*
– *plastic packaging reusable*
– *plastic packaging repurpose*
– *plastic packaging zerowaste*
– *plastic packaging zero-waste*
– *plastic packaging “zero waste”*
– *plastic packaging refill*
– *plastic packaging return*
– *plastic packaging bottle*
– *plastic packaging container*
– *plastic packaging carton*
– *plastic packaging tray*
– *plastic packaging lid*
– *plastic packaging wrap*
– *plastic packaging wrapping*
– *plastic packaging packet*
– *plastic packaging bin*
– *plastic packaging rubbish*
– *plastic packaging trash*
– *plastic packaging landfill*
– *plastic packaging eco*

### Appendix C: Ad hoc reference corpus details

**Table:**

Subcorpus description	Files	Tokens
Around 49,000 UK-geotagged tweets between 2010 and 2021 that mention *yoghurt* or *yoghurts*	1	823,796
Online UK newspaper articles and reader comments on the topic of opera	40	132,007
UK-based focus group transcripts from the ‘People’, ‘Products’, ‘Pests’ and ‘Pets’ project on the subject of animals	19	228,927
**Total**	**60**	**1,184,730**

### Appendix D: Truncated wordlists for Corpus 1 and 2.

**Table:**

	Corpus 1		Corpus 2	
Rank	Frequency	Word	Frequency	Word
1	62,539	*the*	143,767	*the*
2	49,291	*and*	113,275	*to*
3	43,669	*to*	98,400	*i*
4	37,189	*of*	95,930	*and*
5	27,935	*in*	94,951	*a*
6	22,161	*a*	72,100	*of*
7	17,978	*our*	66,623	*plastic*
8	17,201	*we*	66,111	*in*
9	16,717	*for*	64,746	*it*
10	11,800	*is*	47,425	*for*
11	11,277	*on*	45,965	*you*
12	10,632	*are*	40,135	*is*
13	10,445	*with*	38,757	*that*
14	9,193	*s*	35,608	*s*
15	9,095	*as*	33,087	*t*
16	8,768	*be*	32,184	*we*
17	8,152	*that*	31,107	*on*
18	8,104	*by*	31,018	*have*
19	7,785	*from*	27,227	*with*
20	7,471	*can*	26,470	*but*
21	7,378	*or*	26,266	*are*
22	7,247	*at*	24,915	*they*
23	6,783	*this*	23,797	*be*
24	6,654	*plastic*	22,656	*my*
25	6,626	*you*	22,544	*so*
26	6,587	*your*	22,031	*this*
27	6,338	*have*	21,519	*not*
28	6,307	*it*	21,167	*can*
29	6,270	*waste*	21,165	*as*
30	6,121	*recycling*	20,930	*use*
31	5,957	*m*	18,516	*from*
32	5,476	*will*	18,453	*all*
33	5,042	*year*	17,054	*packaging*
34	4,948	*food*	17,008	*just*
35	4,378	*all*	16,573	*if*
36	4,280	*use*	16,568	*your*
37	4,215	*more*	16,415	*at*
38	4,046	*customers*	16,060	*or*
39	4,027	*group*	15,117	*do*
40	3,940	*UK*	14,788	*was*
41	3,786	*which*	14,786	*our*
42	3,782	*not*	14,706	*them*
43	3,714	*financial*	13,538	*up*
44	3,505	*has*	13,504	*waste*
45	3,419	*an*	13,135	*about*
46	3,401	*packaging*	12,926	*out*
47	3,329	*they*	12,770	*more*
48	3,303	*tesco*	12,305	*one*
49	3,157	*their*	12,230	*don*
50	3,134	*also*	11,887	*like*
